# Bi-Objective Flexible Job-Shop Scheduling Problem Considering Energy Consumption under Stochastic Processing Times

**DOI:** 10.1371/journal.pone.0167427

**Published:** 2016-12-01

**Authors:** Xin Yang, Zhenxiang Zeng, Ruidong Wang, Xueshan Sun

**Affiliations:** 1 School of Economics and Management, Hebei University of Technology, Tianjin, China; 2 ZhongHuan Information College Tianjin University of Technology, Tianjin, China; 3 Department of Mathematics, Tianjin University of Technology, Tianjin, China; West Virginia University, UNITED STATES

## Abstract

This paper presents a novel method on the optimization of bi-objective Flexible Job-shop Scheduling Problem (FJSP) under stochastic processing times. The robust counterpart model and the Non-dominated Sorting Genetic Algorithm II (NSGA-II) are used to solve the bi-objective FJSP with consideration of the completion time and the total energy consumption under stochastic processing times. The case study on GM Corporation verifies that the NSGA-II used in this paper is effective and has advantages to solve the proposed model comparing with HPSO and PSO+SA. The idea and method of the paper can be generalized widely in the manufacturing industry, because it can reduce the energy consumption of the energy-intensive manufacturing enterprise with less investment when the new approach is applied in existing systems.

## 1. Introduction

Nowadays, energy efficiency is a very important issue in the world, which is attracting more and more attention from the researchers. As a result, various methods have been proposed to solve the problem [1–6]. In the past 50 years in China, the energy consumption of manufacturing enterprises has accounted for a big proportion of production cost [7]. With the energy prices rising and gradual enhancement of environmental protection awareness, the manufacturing enterprises are facing increasing pressure to reduce energy consumption. According to previously known work, most research has been done on cutting down manufacturing energy consumption by designing more energy efficient machines or improving the machining processes [8]. However, some previously known work also showed this method was not effective enough because the major energy consumed by functions was not directly related to the production of components [9]. Besides, it was implied that there was still a lot of energy consumption margin needed to be reduced at the system-level during the whole production process, so more operational methods needed to be employed as the energy saving approach. Flexible Job-shop Scheduling Problem (FJSP) model was a feasible and effective approach for manufacturing enterprises to reduce the energy consumption, and the machine assignment and the sequencing problem should be solved to satisfy the shortest completion time objective by using the FJSP model.

The actual production process is influenced by many uncertain factors, which makes the FJSP become more complex [10] and some researchers had taken uncertainty into consideration when solving the problem. According to the previous studies, due to the variability of environmental data and the lack of accurate processing models, the processing time of the FJSP was usually uncertain. Based on previous studies, there were usually three methods to describe uncertain factors of the processing times such as random number [11–13], fuzzy number [14–15] and the interval number [16–17]. The application of random number [11–12] and fuzzy number [15] method depended on the large amounts of sample data in advance. However, the large amounts of sample data of the processing times were very difficult to be obtained in practice, and there were limitations in the application process of these methods. Interval number method [17] did not need to consider the specific value or distribution law in the interval, so the applicability and operability of this method seemed better. This method could get a feasible scheduling scheme to a certain extent, but the robustness of the scheduling scheme was not strong and the anti-interference ability to absorb random disturbance in the process was poor. In order to further improve the robustness of the scheduling scheme, Janak et al. [18] proposed a new robust optimization method for kinds of uncertain factors. Tang et al. [19] introduced two uncertain parameters to describe the fluctuation degree and the allowable violation degree of stochastic processing times, and successfully applied the robust optimization method for the single-objective FJSP.

Besides the impact of uncertain factors, the FJSP also belongs to a kind of multi-objective optimization problem. Fattahi P et al. [20] proposed a mathematical model to get an optimization solution. Ozguven C et al. [21] developed a mixed-integer linear programming model for this problem. Models of FJSP proposed by Demir Y et al. [22] included a sequence-position variable-based model, precedence variable-based model and the time indexed model. Furthermore, a multi-objective optimization was a set of Pareto optimal solutions which could be obtained in an optimizing process, and was consistent with an actual scheduling problem. Through the review of the previously known work, the algorithms frequently used to solve FJSP are the artificial intelligence algorithm and its improved algorithms, mainly including: (1) genetic algorithm (GA) and its improved algorithms [23–28], such as the MOGA [29], NPGA [30], NSGA and NSGA-II [31–32] and so on; (2) particle swarm optimization algorithm (PSO) and its improved algorithms [33]. Among them, the NSGA-II algorithm was widely used due to its numerous advantages.

From the review above, it is obvious that most of previous studies considered either the uncertainty problem or the multi-objective FJSP, separately. However, the research on the multi-objective FJSP under uncertain stochastic processing times is relatively rare. The bi-objective FJSP with consideration of the total completion time and the total energy consumption under stochastic processing times is a difficult problem to be solved mainly due to the following issues:

1. How to build the mathematic model of bi-objective FJSP under stochastic processing times. Since the completion time and the total energy consumption are selected as optimal objective and the stochastic processing times should be considered, the mathematic model should be established to consider all these factors.
2. How to propose an effective algorithm for the bi-objective FJSP under stochastic processing times. It is worth noting that the previous algorithms which were designed for solving a single objective easily converged to local optimization. In addition, the performance of the proposed algorithm should also be considered in order to get high quality and many more solutions while spending less time to deal with stochastic process.

**Fig 1:**
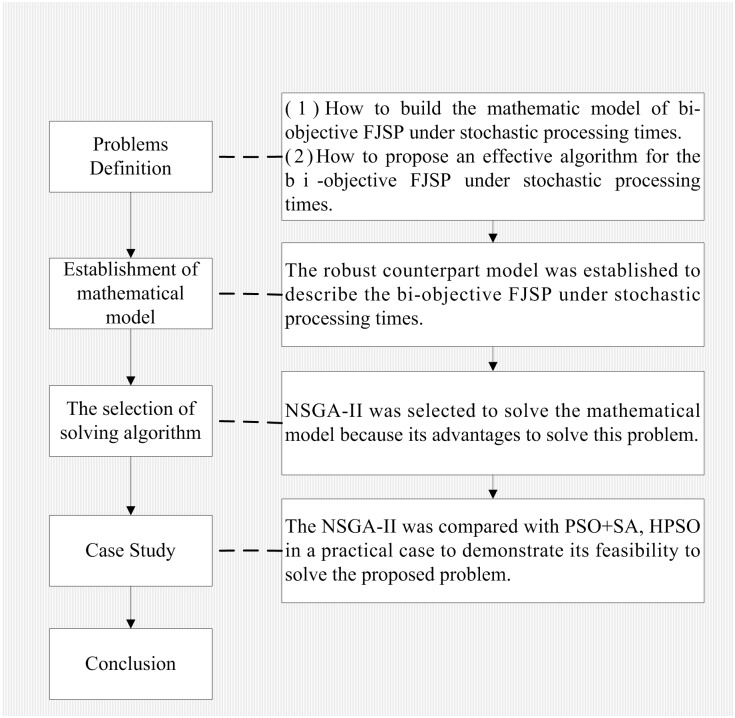
The graphical research framework of this paper.

In this paper, the robust counterpart model and NSGA-II algorithm are used to solve bi-objective FJSP with consideration of the completion time and the total energy consumption under stochastic processing time. Then the feasibility and advantages of the method should be verified while comparing it with conventional methods by experiment and the results. A graphical research framework for this paper has been shown in Fig 1.

## 2. Model and Algorithm

### 2.1 The Mathematic Model of Bi-objective FJSP under Stochastic Processing Time

Bi-objective FJSP under stochastic processing times can be described as: there are jobs and machines, each job contains multiple processes, the sequence of each process is determined, and each process can be processed on a number of different processing machines; the processing time of each job on each machine is different, and the processing time can be changed in a certain range, which must satisfy a certain probability distribution. The efficient solution of the problem, also known as robust scheduling, is achieved by assigning appropriate machines to each process, determining the execution order of each process on each machine such that certain objectives are optimized, with the behavior not affected by random factors.

The notations and assumptions used in the problem statement and throughout the paper are listed as follows:

Notations:

*i*: Job number; *(i = 1*,*2*,*……*,*n)*

*j*: Process number; *(j = 1*,*2*,*……*,*s)*

*k*: Machine number; *(k = 1*,*2*,*……*,*m)*

*t*: Event point; *(t = 1*,*2*,*……*,*m)*

*O*_*ij*_: The *j*^*th*^ process of job *i*;

*M*_*ij*_: The finite machine set of *O*_*ij*_;

*P*_*ijk*_: The nominal processing time of *O*_*ij*_ on machine *k*;

${\tilde{P}}_{ijk}$: The true processing time of *O*_*ij*_ on machine *k*;

*MAX*: A large enough real number;

*S*_*kt*_: Starting time of machine *k* on event point *t*;

*F*_*kt*_: Finishing time of machine *k* on event point *t*;

*ST*_*ij*_: Starting time of *O*_*ij*_;

*FT*_*ij*_: Finishing time of *O*_*ij*_;

*E*_*total*_: The total energy consumption of the whole production process. The calculation of the *E*_*total*_ can be found in S1 Fig and S1 Table.

Assumptions:

1. The processing plan is determined, and all jobs have the same priority;
2. All the machines are available at the beginning time;
3. Each machine can only process one job at a time;
4. Processing cannot be interrupted;
5. Each process has a number of optional processing machines, and the nominal value of processing time on the optional machine is known, but the true value of processing time is obtained from the nominal value by random fluctuation.

There are two kinds of decision making, machine allocation and sequence of operations on each machine. To this end, the variable *Z*_*ijk*_ is introduced. When *Z*_*ijk*_ *= 1*, the *j*^*th*^ process of the *i*^*th*^ job is allocated to the *k*^*th*^ machine at the *t*^*th*^ event point; otherwise *Z*_*ijk*_ = 0. The event point *t* is used to order each process on one machine. Therefore, the mathematical model of the bi-objective FJSP under stochastic processing times is as follows:

$$\begin{array}{l} C_{m}=minC_{max}\\ E_{m}=minE_{total} \end{array}\}$$ (1)

s.t.

$$\sum_{k\in M_{ij}}\sum_{t}Z_{ijkt}=1\;\forall i,j$$ (2)

$$\sum_{i}\sum_{j}Z_{ijkt}\leq 1\;\forall k,t$$ (3)

$$\sum_{i}\sum_{j}Z_{ijkt}\geq \sum_{i}\sum_{j}Z_{ijk,t+1}\;\forall k,t<r$$ (4)

$$F_{kt}\leq S_{k,t+1}\;\forall k,t<r$$ (5)

$$F_{kt}=S_{kt}+\sum_{i}\sum_{j}\left({\tilde{P}}_{ijk}\times Z_{ijkt}\right)\;\forall k,t$$ (6)

$$FT_{ij}\geq FT_{i,j-1}+\sum_{k\in M_{ij}}\sum_{t}\left({\tilde{P}}_{ijk}\times Z_{ijkt}\right)\;\forall i,j>1$$ (7)

$$FT_{ij}\leq FT_{kt}+MAX\times \left(1-Z_{ijkt}\right)\;\forall i,j,k\in M_{ij},t$$ (8)

$$FT_{ij}\geq FT_{kt}-MAX\times (1-Z_{ijkt})\;\forall i,j,k\in M_{ij},t$$ (9)

$$C_{max}\geq F_{kt}\;\forall k,t=r$$ (10)

Formula (1) is the two objective functions of the problem which means to minimize the total completion time and the total energy consumption. The model is subject to the constraints (2) to (10) to ensure its integrity. Constraint (2) ensures the uniqueness of the machine allocation, that is, any process needs and only needs to be allocated to a machine. Constraint (3) limits any machine at any time to perform one process at most. Constraint (4) strictly indicates that the machine must be sorted in the previous order. Constraints (5)–(9) ensure that the processes of each machine are in accordance with the time sequence of orderly implementation, including two aspects: (1) after the completion of a task on the machine, a new task can be started (constraint (5)); (2) once a task is started, it is not allowed to be interrupted (constraint (6)) and the next process can only be started after the completion of the last process (constraint (7)). Constraints (8)–(10) calculate the maximum completion time of a batch of jobs and constraints (8) and (9) establish the one-to-one relationship between the process' completion time and the machine's event point. Constraint (10) defines the completion time constraint, indicating the maximum value of all the machines' finishing time of the last task. It is emphasized that the processing time in the above constraints (6) and (7) is a random variable, which cannot be solved by the conventional deterministic method, so the robust optimal scheduling method needs to be further studied to solve this problem.

In order to generalize this research, the robust counterpart model is established for the bi-objective FJSP under stochastic processing times. The specific transformation process of the bi-objective FJSP model under stochastic processing times into the robust counterpart model with the processing time subjected to the uniform distribution can be found in S1 File.

### 2.2 The NSGA-II Algorithm

NSGA-II is one of the most efficient evolutionary algorithms to solve multi-objective problem. As an improvement of conventional GA, it has the following advantages:

1. The non-dominated sorting operator is proposed in NSGA-II to reduce the computational complexity. According to the previous study in a large number of experimental results [34], the improved fast sorting method is adopted in this paper to shorten the operation time and make more intensive use of CPU resources, which especially has more advantages in solving robust problems.
2. The individual crowding distance operator is used in NSGA-II as the standard of individual comparison in population, so that the individual in the quasi Pareto domain can be extended to the whole Pareto domain to ensure the diversity of the population. At present, the commonly used strategies are niche technology, information entropy, density based clustering, grid and so on. In this paper, the density based clustering strategy is adopted to effectively characterize the distribution and diversity of the population and to maintain the high diversity as well.
3. The elite strategy selection operator is introduced in NSGA-II to enlarge the sampling space. Besides, the parent population and the offspring population is combined together to ensure that some excellent individuals will not be discarded in the evolution in order to improve the accuracy of the optimization results.

**Fig 2:**
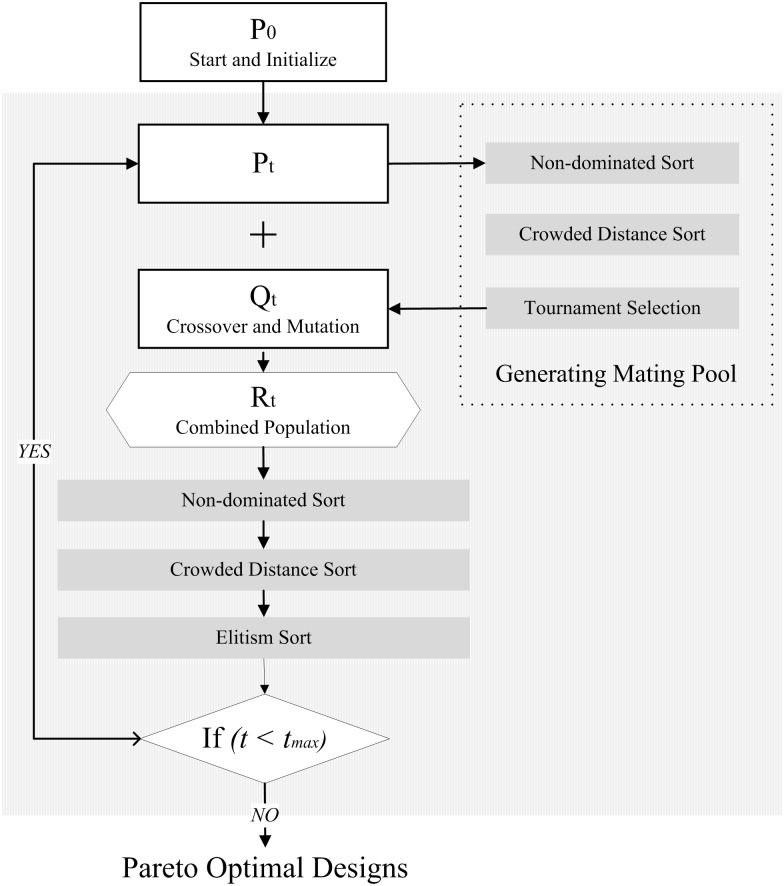
The process of NSGA-II.

Above all, because of the advantages of NSGA-II in solving the multi-objective programming problem, it is considered that NSGA-II still has the applicability in solving the multi-objective FJSP under stochastic processing times. In this paper, the algorithm is applied to the specific case to demonstrate its feasibility. The process of NSGA-II to solve the bi-objective FJSP under stochastic processing times has been shown in Fig 2.

## 3. Case Study

### 3.1. Background

GM Corporation is an equipment-manufacturing enterprise. Its main products include transportation equipment and coal mining equipment. Currently, because the market of selling coal mining equipment has become tight, GM Corporation encounters order reduction. Moreover, under the pressure of energy-saving and emission-reduction policies in China, it has become very urgent to solve the energy-saving problem in GM Corporation. Therefore, the forging job shop in GM Corporation is selected for the case analysis in this paper, because it is the greatest energy consuming job shop in the corporation.

There are several kinds of equipments used in the forging job shop, and different types of equipments are used to complete the same processing function with different energy consumption. According to the data of assembly line in forging job shop, a FJSP is consisted with 10 machines and 6 jobs. And each job goes through 6 operations, including feeding, heating, forging, heat treatment, repair grinding and testing. The data required for analysis of the bi-objective FJSP under stochastic processing time is from the investigation and research of the Science-technology Support Plan of Hebei Province in China, and can be found in S2–S5 Tables.

### 3.2. Analysis and Discussion

The applied algorithm is essential to be considered, in this section, the feasibility of the model and NSGA-II algorithm in solving the multi-objective FJSP under stochastic processing times is verified by experiments and the results. Firstly, the specific implementation process of the NSGA-II algorithm is introduced and the optimal solution as a standard is obtained, then the sensitivity testing for the parameters to further test the model’s performance on two objectives is carried out.

After that, there is an experimental comparison of the proposed algorithm with previous algorithm to conclude the feasibility and advantages of the robust counterpart model and the NSGA-II. Comparing with HPSO and PSO+SA, NSGA-II algorithm is better than these two algorithms in terms of the high quality and many more solutions while spending less operation time under stochastic processing times.

#### 3.2.1 Process of the algorithm and the results

In this section, the NSGA-II can be used to solve the proposed model. The related parameters of the algorithm are set as follows: the population size is 50, the maximum evolution generation is 100, the crossover rate is 100% and the mutation rate is 10%. The encoding schema used in this paper is the two-level encoding and decoding based on procedure and machine which is proposed by Tang [19]. The steps of the NSGA-II to solve the model are as follows:

1. Initial parent population *P*_*0*_ with the size *N* is generated randomly, and the fast non-dominated sorting is implemented on *P*_*0*_.
2. Individual with each rank is sorted again based on the density based clustering strategy to evaluate the population density.
3. Individuals selected are implemented by the binary tournament operator in the mating pool. And between two individuals, the selected individual is the one with the lower rank. If two individuals are on the same level, the winner is the one with the larger value in the crowding distance.
4. The offspring population *Q*_*t*_ is generated by the genetic operations such as crossover and mutation where *“t”* denotes the number of generations in the mating pool. In this paper, the crossover and mutation operators are defined referring to Tang [13].
5. An integrated population *P*_*t*_ is created by combining *P*_*t*_ and *Q*_*t*_, and fitness values are designed to all individuals by the non-dominated sorting and crowded distance sorting.
6. Finally, individuals with better fitness are selected by elitist sorting and these become the parent individuals *P*_*t+1*_.
7. Steps 2–6 are repeated until *t = t*_*max*_. In this case, *t*_*max*_ = 100.
8. Individuals with rank 1 among parents at *P*_*max*_ are pareto-optimal solutions.

After the operation of the NSGA-II according to the data from S2–S5 Tables, the optimal solution of the original problem could be obtained, that is *f*_*1*_ *= 44 (min)*, *f*_*2*_ *= 250*.*8 (kwh)*, which is used as basic scenario for the subsequent parameter sensitivity analysis.

#### 3.2.2. Parameter sensitivity analysis

**Fig 3:**
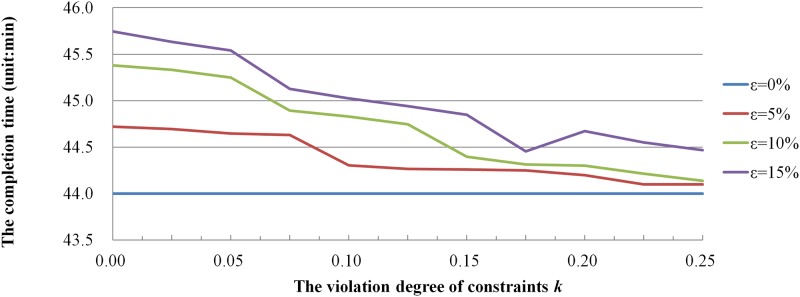
The influence of uncertain parameters on the completion time.

**Fig 4:**
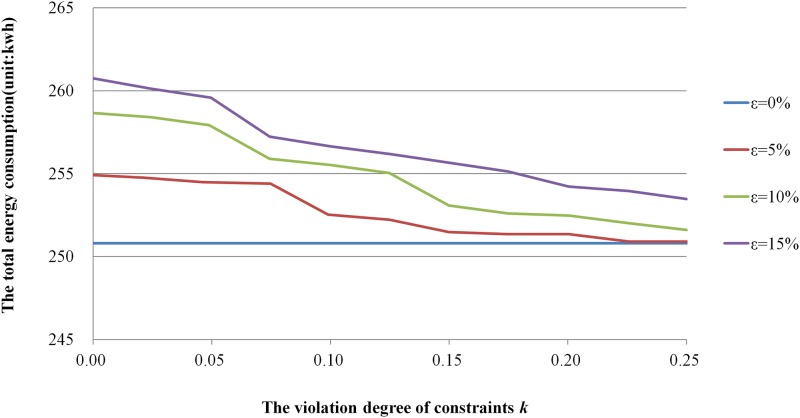
The influence of uncertain parameters on the total energy consumption.

This analysis involved changing the values of k and ε to observe the change of the average values of the completion time and total energy consumption. k represents the allowable violation degree of constraints, and ε (ε>0) is a given uncertainty level to control the fluctuation degree of the uncertain variables. The disturbance of the processing time is subject to [0, 1] uniform distribution. The value of k is taken in the range of 0%~25%evenly, and the value of ε is taken as 5%, 10%, 15% respectively. When the values of k and ε change, the average values of the completion time and total energy consumption is found with 10 runs as shown in Figs 3 and 4.

Through the analysis of the curves in Figs 3 and 4, optimal solution value of the robust counterpart problem is larger than that of under deterministic conditions, and that is because the robust scheduling scheme can more effectively absorb random disturbances of the processing time. The values of two objective functions are steadily increasing with the increase of *ε*, which means the fluctuation degree of stochastic processing times has great impact on the value of two objective functions. However, the values of them gradually decrease with the increase of *k*, which means there is a tradeoff between the allowable violation degree of constraints and the performance loss of scheduling scheme.

In addition, when *ε* decreases with the increase of *k*, the optimal solution value of the robust counterpart problem under uncertain conditions is close to the original problem under deterministic conditions, then less redundancy is need to absorb random disturbances and the performance loss of scheduling scheme is minimal. For example, when *ε = 0*.*15 k = 0*, the values of objective functions of the robust counterpart problem are much higher than these of the original problem under deterministic conditions, it shows that much redundancy is need to absorb random disturbance to ensure the feasibility and resulting in large performance loss of the scheduling scheme.

#### 3.2.3. Algorithm feasibility analysis

In section 3.2.2, the feasibility of the algorithm is verified in terms of the high quality and many more solutions. Because the PSO and its improved algorithm is usually the common algorithm to solve the FJSP problem, the NSGA-II is compared with HPSO and PSO+SA in this paper. The results are shown in Table 1. f_*1*_, f_*2*_ are the objective functions, C_*1*_, C_*2*_ and t_*1*_, t_*2*_ (unit: second) are the solutions and the needed time of the deterministic problem and the robust counterpart problem with ε = 0.1 and k = 0.15 respectively, -t_*1*,_ -t_*2*_ (unit: second) represents the average time to run 10 times in a row.

**Table 1:** Comparison of case results.

Number of times	Deterministic problem					Robust counterpart problem with the parameter (*ε* = 0.1 and *k* = 0.15)		
	PSO+SA	HPSO	*C*_*1*_			*C*_*2*_		
	*f*_*1*_*/f*_*2*_	*f*_*1*_*/f*_*2*_	*f*_*1*_*/f*_*2*_	*t*_*1*_	Average time of *t*_*1*_	*f*_*1*_*/f*_*2*_	*t*_*2*_	Average time of *t*_*2*_
1	(45/248.6)	(45/248.8)	(47/225.8)	9.26	9.34	(47.3/226)	9.48	9.48
			(45/248.2)			(45.2/248.5)		
	(46/236.4)	(44/250.8)	(46/236.1)			(46.6/238)		
			(44/250.8)			(45/251.1)		
2	(45/248.5)	(44/251.2)	(47/225.5)	9.31		(47.6/226.5)	9.52	
			(46/237.1)			(46.3/237.5)		
	(47/226.2)	(46/237.1)	(45/248.2)			(46.1/248.3)		
			(44/251)			(44.2/251.8)		
3	(45/248.3)	(45/249.1)	(47/225.5)	9.44		(47.8/226.5)	9.73	
			(46/237.1)			(46.4/237.8)		
	(44/250.9)	(47/226.8)	(45/248.3)			(46.1/248.3)		
			(44/250.8)			(44.8/252.3)		
4	(45/248.6)	(46/237.4)	(47/226.5)	9.32		(48.2/227.8)	9.54	
			(46/236.7)			(47.1/238.7)		
	(46/236.8)	(44/251.8)	(45/248.2)			(46.8/250.2)		
			(44/251)			(44.6/253.1)		
5	(45/248.3)	(45/248.5)	(47/225.5)	9.22		(47.8/226.5)	9.45	
			(46/237.2)			(46.5/238.2)		
	(44/251)		(45/248.2)			(46.1/249.2)		
			(44/251)			(44.3/251.6)		
6	(46/247.1)	(46/247.3)	(47/225.5)	9.18		(48.1/225.8)	9.34	
			(45/248.1)			(45.6/248.5)		
			(44/250.9)			(44.3/251.4)		
7	(45/248.5)	(45/248.5)	(46/237.1)	9.44		(46.6/238.1)	9.52	
			(45/248.5)			(46.3/248.7)		
			(44/251.2)			(44.2/251.8)		
8	(46/237.2)	(46/238.8)	(47/225.5)	9.34		(47.6/225.9)	9.48	
			(46/237.2)			(46.8/238.2)		
	(44/251)		(45/247.6)			(45.4/248.6)		
			(44/250.8)			(45.1/251.2)		
9	(45/248.5)	(46/237.1)	(47/225.5)	9.21		(48.3/226.5)	9.28	
		(44/252)	(46/237.1)			(47.8/239.1)		
			(44/251.2)			(45.9/253.2)		
10	(45/248.4)	(47/225.6)	(47/225.3)	9.17		(48.9/226.1)	9.43	
			(46/237.1)			(47.2/240.5)		
			(45/248.2)			(46.7/249.2)		

At the environment of huge amount of data, this algorithm is also effective with the tests.

As shown in Table 1, it can be found that the quality of the non-dominated solutions obtained by NSGA-II is better or equal than that of PSO+SA and HPSO, and the quantity of the non-dominated solutions is also more than that of others. It means that the elite strategy selection operator of NSGA-II can ensure that the excellent individuals can be preserved in the evolution, so as to improve the accuracy of the optimization results. Moreover, the density based clustering strategy is used in the calculation of individual crowding distance, and it can effectively maintain the high diversity of the population and generate more non-dominated solutions. In solving the robust counterpart problem, if the processing time has much uncertain disturbance, the operation time of the proposed algorithm is just slightly longer than that of under deterministic conditions, indicating that NSGA-II can quickly and effectively deal with uncertain disturbance.

So the NSGA-II has more advantage when it is used to deal with the increase of operation time caused by the stochastic processing times, to ensure the operation time of FJSP under stochastic processing times is acceptable. The NSGA-II is feasible to solve bi-objective FJSP with consideration of the total completion time and the total energy consumption under stochastic processing times.

## 4. Conclusion and Future Work

The bi-objective FJSP with consideration of the total completion time and the total energy consumption under stochastic processing times is a difficult problem to solve because of the complexity of this problem. For this problem, in this paper a robust counterpart model assuming the processing time which is subjected to the uniform distribution is established, and then NSGA-II is used to solve the model. By the case study and comparing with PSO+SA and HPSO, the results all can prove that the NSGA-II is feasible and has advantages to solve bi-objective FJSP with consideration of the completion time and the total energy consumption under stochastic processing times. As for further research directions, reducing the energy consumption in a dynamic flexible job shop should be further studied.

## Supporting Information

S1 Fig The status of equipment and corresponding energy distribution curve. (DOC)

S1 File The specific transformation process of the bi-objective FJSP model under stochastic processing times. (DOC)

S1 Table The parameters of the energy consumption of the specific machine. (DOC)

S2 Table Optional machine set for working procedure. (DOC)

S3 Table Processing time on different machines (unit: min). (DOC)

S4 Table The average energy consumption per working procedure on different machines (unit: kw). (DOC)

S5 Table Other parameters of the energy consumption of the specific machine. (DOC)
