# Supplementary material for: Bi-Objective Flexible Job-Shop Scheduling Problem Considering Energy Consumption under Stochastic Processing Times
Source: PLoS One. 2016 Dec 1;11(12):e0167427. doi: 10.1371/journal.pone.0167427 (PMC5131930; doi:10.1371/journal.pone.0167427)
Supplement: S1 Fig — (DOC) [file pone.0167427.s001.doc]

Supporting Information

**Bi-objective Flexible Job-shop Scheduling Problem Considering Energy Consumption under Stochastic Processing Times**

Xin Yang^1,2^,*, Zhenxiang Zeng^1^,*, Ruidong Wang^3^, Xueshan Sun^2^

**1** School of Economics and Management, Hebei University of Technology, Tianjin, China

**2** ZhongHuan Information College Tianjin University of Technology, Tianjin, China

**3** Department of Mathematics, Tianjin University of Technology, Tianjin, China

*** Corresponding Author**

**E-mail:** [**wing.lps@163.com**](mailto:wing.lps@163.com) **(XY),** [**xzeng@hebut.edu.cn**](mailto:xzeng@hebut.edu.cn) **(ZXZ)**

The calculation of the total energy consumption of the whole production process

Machinery processing equipment’s status include: start-up, shut-down, no-load and processing. Different status presents different energy consumption characteristics. After a large number of experiments, the general status of equipment and corresponding energy distribution curve is displayed in S1 Fig. The energy consumption of the specific machine can be represented by the following parameters in S1 Table.


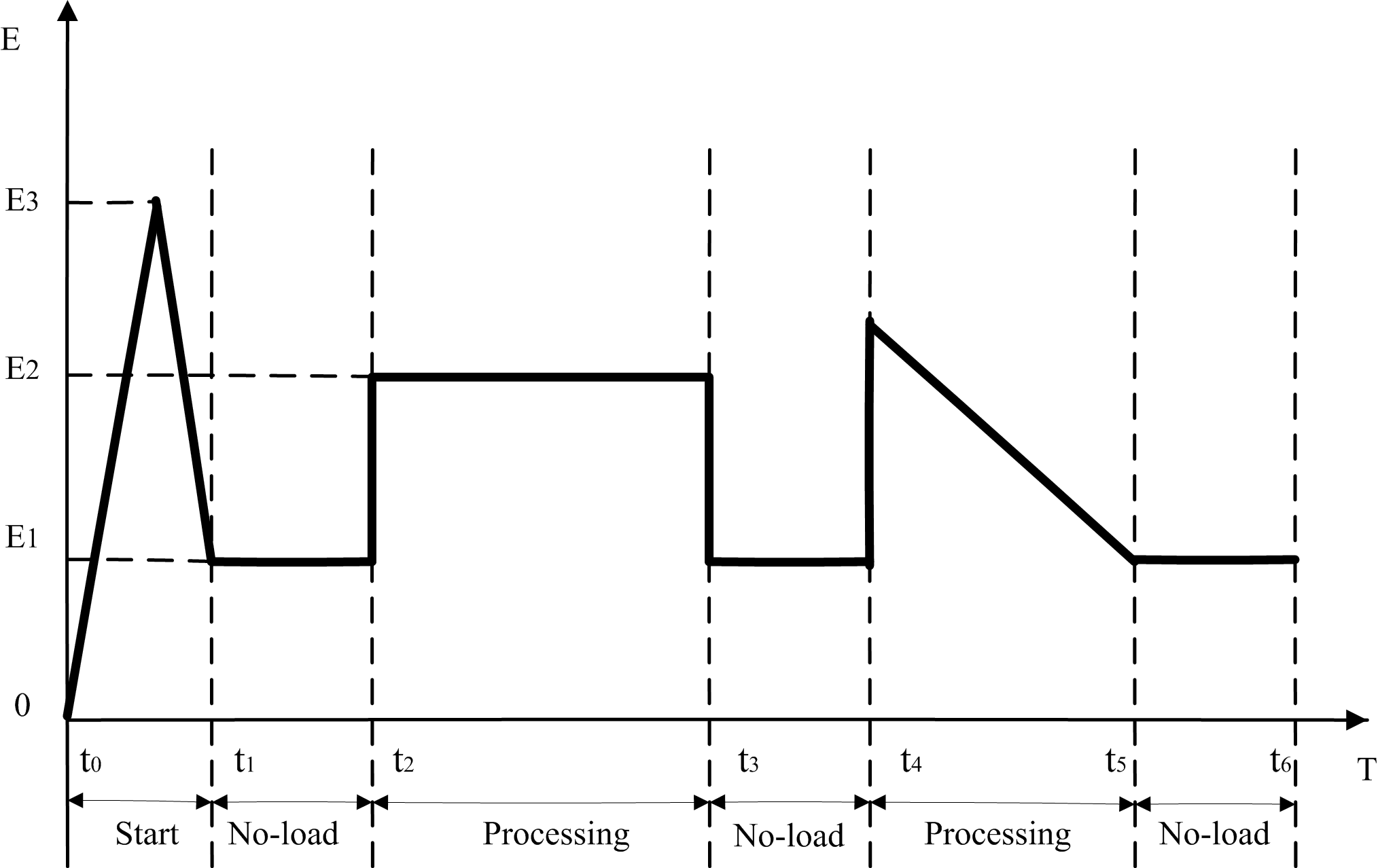


S1 Fig. The status of equipment and corresponding energy distribution curve

So, $E_{total}=\sum E_{s}+\sum E_{b}+\sum E_{e}$ indicates the total energy consumption of the whole production process, which is the sum of all machines' energy consumption in the whole process of production. Therefore, after the completion of the processing task, in order to minimize the total energy consumption, the following aspects must be considered:

(1) Different machines undertake different processes, so the energy consumption is also variable.

(2) Assuming that there is a time interval *T_v_* between different processes at the machine *k*, the use of no-load or shut-down would directly affect the consumption of energy, given by$T_{v}\leq\overline{e_{s}^{k}}\bullet t_{s}^{k}/\overline{e_{e}^{k}}$, if using no-load running, and given by $T_{v}>\overline{e_{s}^{k}}\bullet t_{s}^{k}/\overline{e_{e}^{k}}$, if using shut-down.
