# Supplementary material for: Bi-Objective Flexible Job-Shop Scheduling Problem Considering Energy Consumption under Stochastic Processing Times
Source: PLoS One. 2016 Dec 1;11(12):e0167427. doi: 10.1371/journal.pone.0167427 (PMC5131930; doi:10.1371/journal.pone.0167427)
Supplement: S1 File — (DOC) [file pone.0167427.s002.doc]

Supporting Information

**Bi-objective Flexible Job-shop Scheduling Problem Considering Energy Consumption under Stochastic Processing Times**

Xin Yang^1,2^,*, Zhenxiang Zeng^1^,*, Ruidong Wang^3^, Xueshan Sun^2^

**1** School of Economics and Management, Hebei University of Technology, Tianjin, China

**2** ZhongHuan Information College Tianjin University of Technology, Tianjin, China

**3** Department of Mathematics, Tianjin University of Technology, Tianjin, China

*** Corresponding Author**

**E-mail:** [**wing.lps@163.com**](mailto:wing.lps@163.com) **(XY),** [**xzeng@hebut.edu.cn**](mailto:xzeng@hebut.edu.cn) **(ZXZ)**

S1 File. The specific transformation process of the bi-objective FJSP model under stochastic processing times

According to the mathematical model of the bi-objective FJSP under stochastic processing times in the paper, the equality constraints in relaxation formula (6) in the manuscript could be defined as:

(1)

Where, *φ* is small enough to be real. Additionally formula (7) in the manuscript can be represented by formula (2),

(2)

For the convenience of research, the formula (1)-(2) can be changed to formula (3) shown as a general expression, in which *x* is a continuous variable, *y* is 0 or 1, *A*, *B* is the coefficient matrix, and *P* is the column vector. Here, the parameters' uncertainty refers to the uncertainty which can exist in *A*, *B* or *P*.

$Ax+By\leq P$ (3)

Taking into consideration of the uncertain variables’ fluctuation around the nominal values, the uncertain variables *A*, *B* and *P* can be represented by the nominal value and the stochastic value as shown in formula (4).

$\widetilde{a}=(1+\varepsilon\xi_{a})a$ $\widetilde{b}=(1+\varepsilon\xi_{b})b$ $\widetilde{p}=(1+\varepsilon\xi_{p})p$ (4)

In formula (4), , and are the true values of the uncertain coefficients *A*, *B* and *P*. *ξa*, *ξb* and *ξp* represent the independent fluctuation degree of the uncertain variables , and . *ε (ε>0)* is a given uncertainty level to control the fluctuation degree of the uncertain variables. It can be seen that the greater the value of *ε*, the greater the fluctuation degree of stochastic processing times, the smaller possibility to satisfy the constraints, the worse capacity and narrow scope of scheduling scheme to absorb random disturbances under specified robustness level.

In order to achieve a tradeoff between allowable violation degree of constraints and the scheduling schema, the tradeoff parameter *k* is proposed to represent the allowable violation degree of constraints, hence *1-k* represents the establishment possibility of the inequality. The solution can be called the robust solution when the allowable violation degree of constraint is *k*. Assuming that *ξa*, *ξb* and *ξp* is a random variable subjected to *[−1, 1]* uniform distribution, its distribution function is,

$F(\xi)=\frac{\xi-(-1)}{1-(-1)}=\frac{\xi+1}{2},(-1\leq\xi\leq1);and \text{F}^{-1}(\xi)=2\xi-1,(0\leq\xi\leq1)$ (5)

According to formula(4), the relationship between the true processing time and the nominal processing time *p* could be described by the fluctuation degree of random variable *ε* and the processing time disturbance *ξ*:

(6)

In summary, the bi-objective FJSP under stochastic processing times can be transformed into the robust counterpart model with the processing time subjected to the uniform distribution.
