# Supplementary material for: Bi-Objective Flexible Job-Shop Scheduling Problem Considering Energy Consumption under Stochastic Processing Times
Source: PLoS One. 2016 Dec 1;11(12):e0167427. doi: 10.1371/journal.pone.0167427 (PMC5131930; doi:10.1371/journal.pone.0167427)
Supplement: S1 Table — (DOC) [file pone.0167427.s003.doc]

Supporting Information

**Bi-objective Flexible Job-shop Scheduling Problem Considering Energy Consumption under Stochastic Processing Times**

Xin Yang^1,2^,*, Zhenxiang Zeng^1^,*, Ruidong Wang^3^, Xueshan Sun^2^

**1** School of Economics and Management, Hebei University of Technology, Tianjin, China

**2** ZhongHuan Information College Tianjin University of Technology, Tianjin, China

**3** Department of Mathematics, Tianjin University of Technology, Tianjin, China

*** Corresponding Author**

**E-mail:** [**wing.lps@163.com**](mailto:wing.lps@163.com) **(XY),** [**xzeng@hebut.edu.cn**](mailto:xzeng@hebut.edu.cn) **(ZXZ)**

The calculation of the total energy consumption of the whole production process

S1 Table The parameters of the energy consumption of the specific machine

| Parameters | Description |
| --- | --- |
| $\overline{e_{s}^{k}}$ | the average energy consumption of the $k^{th}$ machine at start-up stage |
| $t_{s}^{k}$ | the $k^{th}$ machine's start-up time |
| $E_{s}^{k}=\overline{e_{s}^{k}}\times t_{s}^{k}$ | the $k^{th}$ machine's total start-up energy consumption at one time |
| $\overline{e_{e}^{k}}$ | the average energy consumption of the $k^{th}$ machine at no-load stage |
| $\overline{e_{b}^{ijk}}$ | the $k^{th}$ machine's average energy consumption of machining the $i^{th}$ job at the $j^{th}$ process |
| $t_{b}^{ijk}$ | the time consumption of machining the $i^{th}$ job at the $j^{th}$ process |
| $E_{b}^{ijk}=\overline{e_{b}^{ijk}}\times t_{b}^{ijk}$ | the machine's total energy consumption of machining the $i^{th}$ job at the $j^{th}$ process |
