# Supplementary material for: Bi-Objective Flexible Job-Shop Scheduling Problem Considering Energy Consumption under Stochastic Processing Times
Source: PLoS One. 2016 Dec 1;11(12):e0167427. doi: 10.1371/journal.pone.0167427 (PMC5131930; doi:10.1371/journal.pone.0167427)
Supplement: S2 Table — (DOC) [file pone.0167427.s004.doc]

Supporting Information

**Bi-objective Flexible Job-shop Scheduling Problem Considering Energy Consumption under Stochastic Processing Times**

Xin Yang^1,2^,*, Zhenxiang Zeng^1^,*, Ruidong Wang^3^, Xueshan Sun^2^

**1** School of Economics and Management, Hebei University of Technology, Tianjin, China

**2** ZhongHuan Information College Tianjin University of Technology, Tianjin, China

**3** Department of Mathematics, Tianjin University of Technology, Tianjin, China

*** Corresponding Author**

**E-mail:** [**wing.lps@163.com**](mailto:wing.lps@163.com) **(XY),** [**xzeng@hebut.edu.cn**](mailto:xzeng@hebut.edu.cn) **(ZXZ)**

The Data Required for Analysis of the bi-objective FJSP under stochastic processing times in Case Study

S2 Table. Optional machine set for working procedure

| J-m | O_11_ | O_12_ | O_13_ | O_14_ | O_15_ | O_16_ |
| --- | --- | --- | --- | --- | --- | --- |
| Job1 | 5 | 6 | 4 | [2,9] | [3,7] | 5 |
| Job2 | 4 | [2,9] | 8 | [6,7] | 5 | [1,10] |
| Job3 | 3 | [6,8] | 7 | [2,1] | [4,10] | 5 |
| Job4 | 5 | 2 | [4,7] | 10 | [2,5] | [3,6] |
| Job5 | [4,5] | 5 | [9,10] | 6 | 2 | [3,8] |
| Job6 | [2,6] | 4 | [6,9] | 7 | 8 | [3,9] |
