# Supplementary material for: Bi-Objective Flexible Job-Shop Scheduling Problem Considering Energy Consumption under Stochastic Processing Times
Source: PLoS One. 2016 Dec 1;11(12):e0167427. doi: 10.1371/journal.pone.0167427 (PMC5131930; doi:10.1371/journal.pone.0167427)
Supplement: S4 Table — (DOC) [file pone.0167427.s006.doc]

Supporting Information

**Bi-objective Flexible Job-shop Scheduling Problem Considering Energy Consumption under Stochastic Processing Times**

Xin Yang^1,2^,*, Zhenxiang Zeng^1^,*, Ruidong Wang^3^, Xueshan Sun^2^

**1** School of Economics and Management, Hebei University of Technology, Tianjin, China

**2** ZhongHuan Information College Tianjin University of Technology, Tianjin, China

**3** Department of Mathematics, Tianjin University of Technology, Tianjin, China

*** Corresponding Author**

**E-mail:** [**wing.lps@163.com**](mailto:wing.lps@163.com) **(XY),** [**xzeng@hebut.edu.cn**](mailto:xzeng@hebut.edu.cn) **(ZXZ)**

The Data Required for Analysis of the bi-objective FJSP under stochastic processing times in Case Study

S4 Table. The average energy consumption per working procedure on different machines (unit: kw)

| $\overline{e_{b}^{ijk}}$ | O_11_ | O_12_ | O_13_ | O_14_ | O_15_ | O_16_ |
| --- | --- | --- | --- | --- | --- | --- |
| Job1 | 2.5 | 3.1 | 1.2 | [2.3,2.6] | [1.8,3.2] | 2.1 |
| Job2 | 2.2 | [3.2,1.1] | 0.2 | [2.5,3.5] | 2.2 | [2.4,1.9] |
| Job3 | 3.1 | [3.2,2.1] | 2.1 | [3.2,3.1] | [1.5,0.7] | 0.8 |
| Job4 | 2.6 | 3.1 | [2.6,3.4] | 3.5 | [1.3,0.9] | [1.7,2.5] |
| Job5 | [2.5,1.6] | 1.5 | [3.2,1.1] | 2.0 | 2.4 | [2.7,0.9] |
| Job6 | [3.1,1.2] | 2.5 | [2.3,2.6] | 3.5 | 2.2 | [1.5,2.6] |
