# Supplementary material for: Bi-Objective Flexible Job-Shop Scheduling Problem Considering Energy Consumption under Stochastic Processing Times
Source: PLoS One. 2016 Dec 1;11(12):e0167427. doi: 10.1371/journal.pone.0167427 (PMC5131930; doi:10.1371/journal.pone.0167427)
Supplement: S5 Table — (DOC) [file pone.0167427.s007.doc]

Supporting Information

**Bi-objective Flexible Job-shop Scheduling Problem Considering Energy Consumption under Stochastic Processing Times**

Xin Yang^1,2^,*, Zhenxiang Zeng^1^,*, Ruidong Wang^3^, Xueshan Sun^2^

**1** School of Economics and Management, Hebei University of Technology, Tianjin, China

**2** ZhongHuan Information College Tianjin University of Technology, Tianjin, China

**3** Department of Mathematics, Tianjin University of Technology, Tianjin, China

*** Corresponding Author**

**E-mail:** [**wing.lps@163.com**](mailto:wing.lps@163.com) **(XY),** [**xzeng@hebut.edu.cn**](mailto:xzeng@hebut.edu.cn) **(ZXZ)**

The Data Required for Analysis of the bi-objective FJSP under stochastic processing times in Case Study

S5 Table. Other parameters of the energy consumption of the specific machine

| m | M_1_ | M_2_ | M_3_ | M_4_ | M_5_ | M_6_ | M_7_ | M_8_ | M_9_ | M_10_ |
| --- | --- | --- | --- | --- | --- | --- | --- | --- | --- | --- |
| $t_{s}^{k}$(unit: min) | 2 | 1.5 | 2 | 2 | 2.5 | 1 | 2 | 1.5 | 2 | 2 |
| $\overline{e_{e}^{k}}$(unit: kw) | 0.5 | 0.3 | 0.32 | 0.41 | 0.36 | 0.5 | 0.25 | 0.36 | 0.52 | 0.35 |
| $\overline{e_{s}^{k}}$(unit: kw) | 1.52 | 1.61 | 1.83 | 1.5 | 1.2 | 1.55 | 1.67 | 1.9 | 1.36 | 1.56 |
